# Histone hypoacetylation contributes to CXCL12 downregulation in colon cancer: impact on tumor growth and cell migration

**DOI:** 10.18632/oncotarget.16323

**Published:** 2017-03-17

**Authors:** Benoît Romain, Radhia Benbrika-Nehmar, Laetitia Marisa, Michèle Legrain, Viviane Lobstein, Attila Oravecz, Laetitia Poidevin, Cyril Bour, Jean-Noël Freund, Isabelle Duluc, Dominique Guenot, Erwan Pencreach

**Affiliations:** ^1^ Université de Strasbourg, Progression Tumorale et Microenvironnement, Approches Translationnelles et Epidémiologie, Strasbourg, France; ^2^ Hôpitaux Universitaires de Strasbourg, Service de Chirurgie Générale et Digestive, Strasbourg, France; ^3^ Cartes d'Identité des Tumeurs Program, Ligue Nationale Contre le Cancer, Paris, France; ^4^ Hôpitaux Universitaires de Strasbourg, Laboratoire de Biochimie et Biologie Moléculaire, Strasbourg, France; ^5^ Université de Strasbourg, CNRS, Department of Computer Science, ICube, Strasbourg, France; ^6^ Université de Strasbourg, INSERM Unit 1113, Strasbourg, France; ^7^ Hôpitaux Universitaires de Strasbourg, Centre de Ressources Biologiques, Département de Pathologie, Strasbourg, France

**Keywords:** chemokine, valproate, butyrate, PCAF, acetylation

## Abstract

CXCL12 has been shown to be involved in colon cancer metastasis, but its expression level and molecular mechanisms regulating its expression remain controversial. We thus evaluated CXCL12 expression in a large cohort of colon adenomas and carcinomas, investigated for an epigenetic mechanism controlling its expression and evaluated the impact of CXCL12 levels on cell migration and tumor growth. CXCL12 expression was measured in human colon adenomas and carcinomas with transcriptome array and RT-qPCR. The promoter methylation was analyzed with whole-genome DNA methylation chips and protein expression by immunohistochemistry. We confirm a reduced expression of CXCL12 in 75% of MSS carcinomas and show that the decrease is an early event as already present in adenomas. The methylome analysis shows that the CXCL12 promoter is methylated in only 30% of microsatellite-stable tumors. *In vitro*, treatments with HDAC inhibitors, butyrate and valproate restored CXCL12 expression in three colon cell lines, increased acetylation of histone H3 within the CXCL12 promoter and inhibited cell migration. *In vivo*, valproate diminished (65%) the number of intestinal tumors in APC mutant mice, slowed down xenograft tumor growth concomitant to restored CXCL12 expression. Finally we identified loss of PCAF expression in tumor samples and showed that forced expression of PCAF in colon cancer cell lines restored CXCL12 expression. Thus, reduced PCAF expression may participate to CXCL12 promoter hypoacetylation and its subsequent loss of expression. Our study is of potential clinical interest because agents that promote or maintain histone acetylation through HDAC inhibition and/or HAT stimulation, may help to lower colon adenoma/carcinoma incidence, especially in high-risk families, or could be included in therapeutic protocols to treat advanced colon cancer.

## INTRODUCTION

Stromal cell-derived factor-1 (SDF-1 or CXCL12) has been implicated in embryonic development, vasculogenesis, and hematopoietic stem cell engraftment, lymphocyte trafficking and wound healing [1]. This cytokine and its cognate receptors, CXCR4 and CXCR7, are essential, as their knockout during embryogenesis or the perinatal period is lethal [2–4]. In the normal colon epithelium, CXCL12 is highly expressed at the top of the crypt-cuff axis and regulates important physiological mechanisms, through facilitation of ion transport, epithelial cell migration, maintenance of intestinal barrier integrity and restoration [5, 6].

Chemotactic cytokines and their receptors are also involved in cancer and promote tumor initiation, progression and metastasis [7, 8]. Because the metastatic dissemination of malignant cells in target organs is a strong determinant of human mortality, much effort has been devoted to understanding how cytokines participate in this multistep process. Growing evidence of colon cancer *in vivo* indicates that a high expression level of CXCR4 receptor facilitates the non-random extravasation of tumor cells after they have left the primary tumor [9], especially in the liver, lungs or bone marrow, where CXCL12 is highly expressed. In line with this evidence, Wendt et al, recently suggested that DNA hypermethylation results in a loss of CXCL12 expression in tumor cells, which might promote the metastasis of colon and breast cancers by creating an enhanced chemotactic gradient between the CXCL12-poor microenvironment of the primary tumor and the CXCL12-rich target organs [10, 11]. However, the pattern and function of CXCL12 expression remain controversial, because other studies have reported that CXCL12 expression was increased in colon carcinomas or adenomas [12–14].

Considering the conflicting reports on CXCL12 expression in human colorectal cancers as well as the incertitude that surrounds the mechanisms of dysregulation either DNA hypermethylation or/and histone deacetylation [10, 15], we analyzed CXCL12 in a large collection of human adenomas and carcinomas and the gene promoter methylation status from a whole-genome DNA methylation study. The level of CXCL12 expression was also evaluated in the six molecular subtypes (C1 to C6) that we previously identified from a discovery subset of 443 samples based on mRNA expression profile analyses [16]. These subtypes were associated with distinct clinic-pathological characteristics, molecular alterations, and specific enrichments of supervised gene expression signatures and deregulated signaling pathways.

Furthermore, we assessed the respective roles of DNA methylation and histone acetylation in colon cell lines and intestinal tumor-prone mice.

## RESULTS

### CXCL12 expression in human colon cancers based on transcriptome arrays

**Figure 1:**
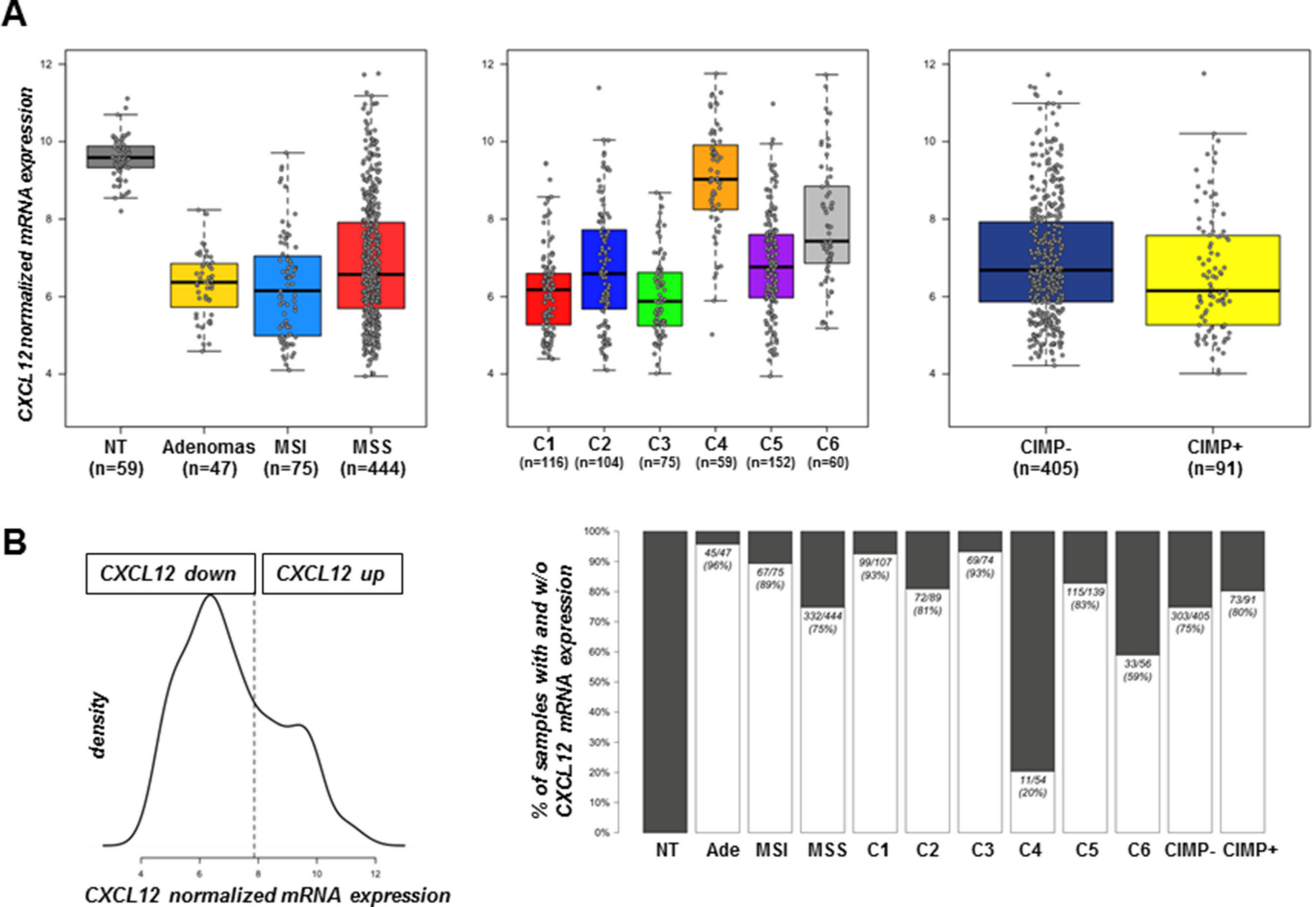
CXCL12 mRNA expression distribution (**A**) Boxplot of intensity values according to sample types (left) (47 adenomas, 75 MSI carcinomas, 444 MSS carcinomas, and 59 non-tumor tissues), colon cancer subtype (middle) (*n* = 566 MSS and MSI carcinomas of the discovery set) and CIMP status (right) (*n* = 496). (**B**) Distribution and discretization of CXCL12 expression established from 702 samples, including the 566 samples (19), 30 carcinomas from the GSE4183 data set, the 59 non-tumor tissues and 47 carcinomas of undefined phenotype. (Left) Density estimation of CXCL12 expression value with mclust approach defining two distinct distributions that correspond to a cut-off value of 7.8. (Right) Proportion of the defined discretization of CXCL12 expression within sample type, colon cancer subtype and CIMP status groups. Black boxes indicate % of samples not expressing CXCL12 mRNA; white boxes indicate the % of samples expressing CXCL12 mRNA.

We analyzed a large cohort of tumor samples that was already used for molecular classification of colon carcinomas [16] as well as 2 series of adenomas. Both MSI and MSS tumors and adenomas expressed 4-fold less CXCL12 than unpaired normal tissues and this difference was significant (*p* < 1e^−16^) (Figure 1A). Our previous work [16] identified six carcinoma subtypes of prognostic value and we show here that CXCL12 expression is decreased in all subtypes, although to a lesser extent in the subtypes C4 and C6 (*p* = 4.4e-03 for C4 and *p* = 1.1e-11 for C6 vs. normal tissue - Figure 1A). The level of CXCL12 transcript was also significantly decreased in adenomas (*p* = 2.2e^−36^), which indicated an early event in the pathological sequence. The unsupervised classification of CXCL12 expression values defined 2 groups of samples with the optimal model, which corresponded to a high CXCL12-expressing group (*n* = 221) that contained 100% of the non-tumor samples and a second group that expressed less CXCL12 (*n* = 481) (Figure 1B). Overall, CXCL12 expression was decreased in 94% (44/47) of the adenomas, 85% (64/75) of the MSI carcinomas and 75% (335/444) of the MSS tumors. As regards the CpG island methylation phenotype, although a slight decrease of expression level was observed for samples with CIMP-High status, no significant association was found between the level of CXCL12 expression groups and the CIMP status (*p* = 0.34).

### Genomic status of CXCL12

The status of the CXCL12 gene locus at 10q11.1 was analyzed in carcinomas using CGH array data on a 4434 BAC-array with a median resolution of 0.6 Mb [16]. Allelic losses and gains were observed at the CXCL12 locus in 52/347 (15%) and 23/347 (7%) of the MSS tumors, respectively; no losses and only 5/69 gains (7%) were observed in the MSI tumors and as exampled on Figure 4 for the methylation cohort, the level of CXCL12 expression was not significantly associated with the gene copy number (*p* = 0.13).

### CXCL12 in human and murine colon cancers assessed by immunohistochemistry and RT-qPCR

**Figure 2:**
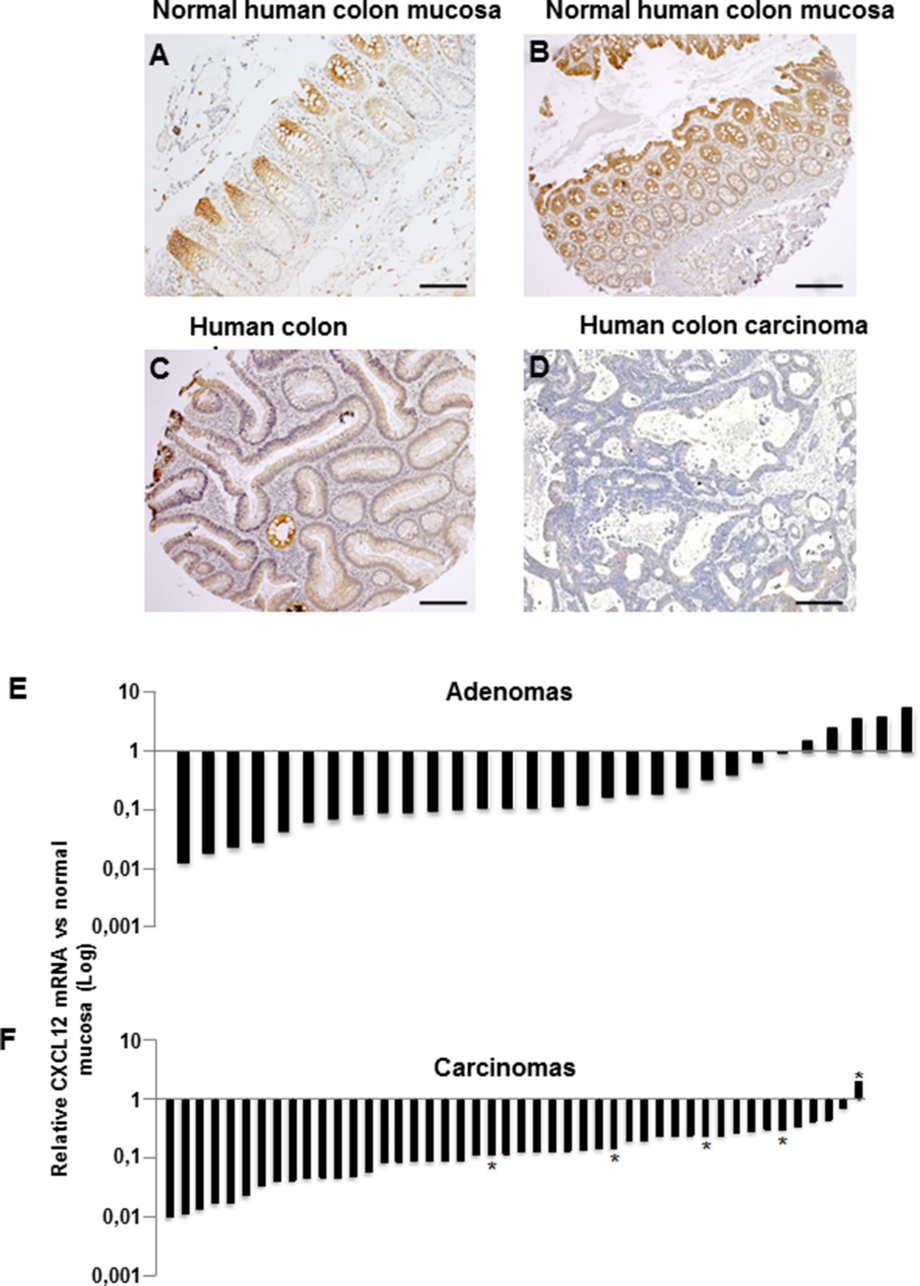
CXCL12 protein and mRNA expression Immunohistochemical expression in (**A**–**B**) normal human colon mucosa, (**C**) an adenoma and a (**D**) carcinoma. (**E–F**) CXCL12 mRNA expression by RT-qPCR in human colon adenomas (E, *n* = 30) and carcinomas (F, *n* = 46). *Liver metastases. All quantifications were performed in duplicate in three independent experiments and normalized to the endogenous PDGF mRNA levels.

CXCL12 expression was analyzed at the protein and mRNA levels in an independent cohort that included 30 colon adenomas and 46 sporadic MSS carcinomas (Table 1). In the normal intestinal mucosa, CXCL12 protein was weakly expressed at the crypt base, and this expression increased along the crypt-surface epithelial cuff axis and was maximized at the epithelium surface (Figure 2A–2B). Corroborating the transcriptomic array data from a national cohort, the CXCL12 expression was strongly decreased in a local cohort of 23/30 adenomas and in 45/46 carcinomas at both the protein (Figure 2C–2D) and mRNA levels (adenomas: mean relative ratio: 0.28; *p* = 0.008; carcinomas: mean relative ratio: 0.18; *p* = 0.005; Figure 2E–2F). The CXCL12 expression in adenomas did not significantly differ by distinct histological subtype or degree of dysplasia.

**Table 1:** Characteristics of the 30 adenomas and 46 carcinomas analyzed for CXCL12 mRNA

Adenomas		*n* = 30	Carcinomas		*n* = 46
***Mean age ± SD***		66,5 ± 10,2	***Mean age***		68,7 ± 12
***Men:Women***		19:11	***Men:Women***		33:13
***Localization***	Proximal colon	15	***Localization***	Proximal colon	21
	Distal colon	15		Distal colon Metastasis	24 1
***UICC classification***	Hyperplasia	0	***UICC classification***	In situ Tis	2
	Villous	1		I	8
	Tubulovillous	3		II	12
	Tubular	26		III	10
				IV	14
***Grade***	Low	23			
	High	7			

Abbreviations: SD, standard deviation.

**Figure 3:**
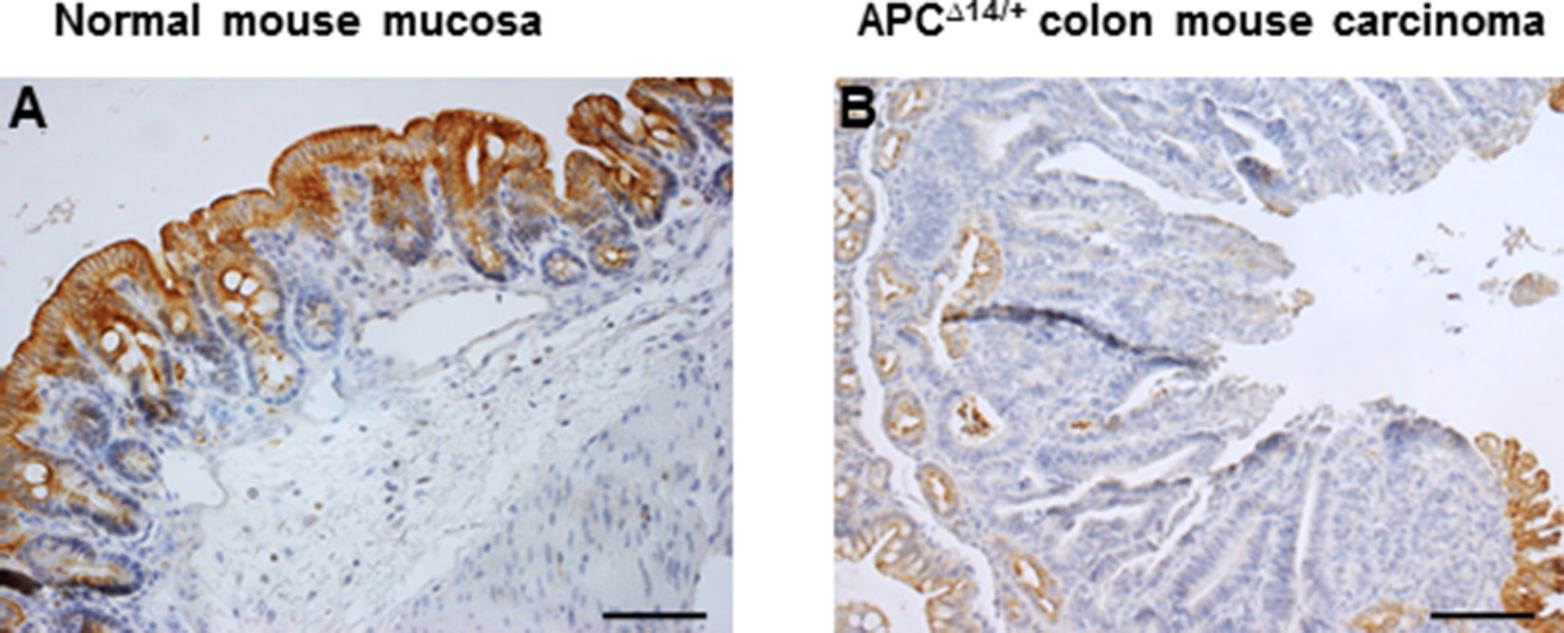
Immunohistochemical expression of CXCL12 in (**A**) normal mouse colon mucosa and (**B**) APC^Δ14/+^ mouse colon carcinoma.

We attempted to demonstrate whether the loss of CXCL12 expression observed in human carcinomas would also be found in mouse models of intestinal tumors. As for human normal colon mucosa, the expression of CXCL12 increased along the crypt-surface epithelial cuff axis in wild-type mice (Figure 3A). Moreover, in intestinal adenomas and adenocarcinomas that occurred in APC^Δ14/+^ (Figure 3B), APC^Min/+^ or azoxymethane-treated wild-type mice (data not shown), CXCL12 expression was strongly reduced.

These results demonstrate that the downregulation of CXCL12 expression is an early event during malignant colon epithelial cell transformation in both humans and mice.

### CXCL12 promoter methylation status in human colon tumors

**Figure 4:**
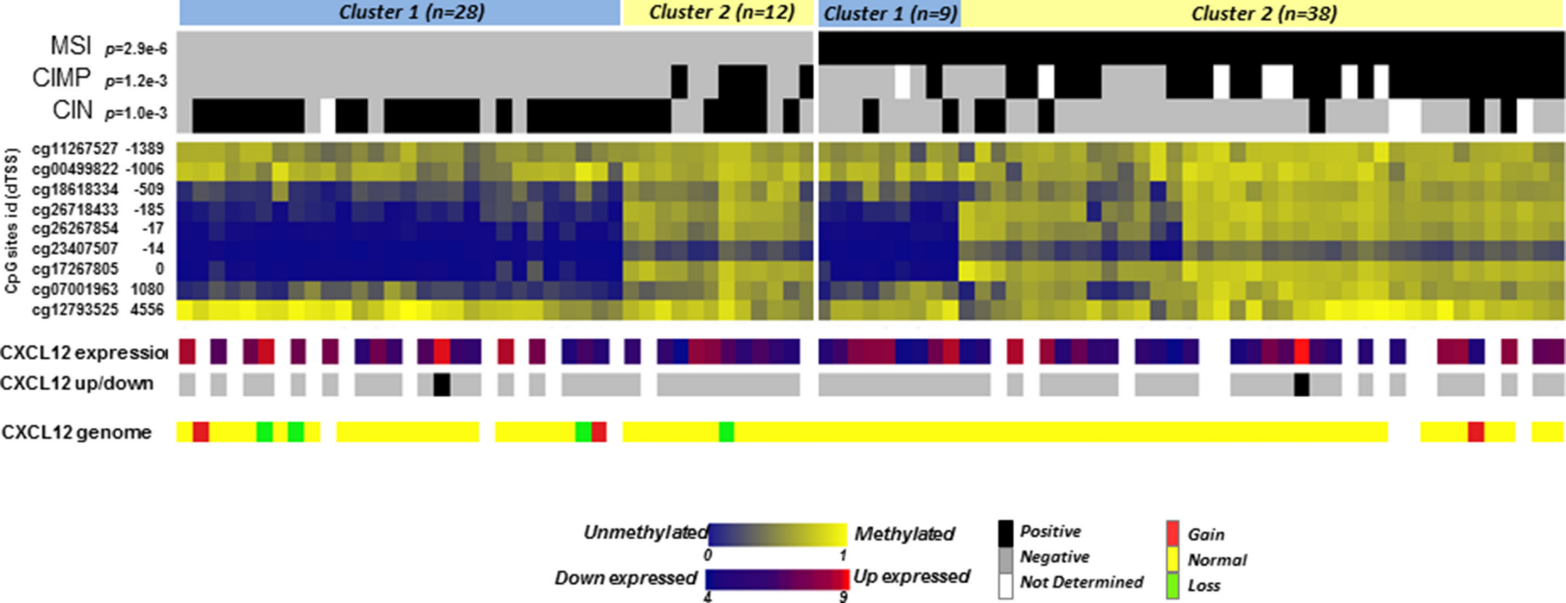
Heatmap of methylation levels of CpG sites in the CXCL12 CpG island region β-values of the 9 CpG sites in the CpG island associated with CXCL12 are represented in blue (low level of methylation) to yellow (high level of methylation). The values are ordered based on clusters separately defined by unsupervised hierarchical clustering in MSS and MSI tumors and by CpG site location distances from the transcription start site (from -1389 to 4556). The molecular annotations of the samples and their *p*-value of association with the cluster defined by Fisher's exact test are given at the top. At the bottom, the expression levels of CXCL12, the recoded up- or downregulated expression group as defined above and the genomic loss/gain of CXCL12 are displayed.

Aberrant gene silencing in tumor cells is often associated with gene promoter methylation, and CpG island methylation has been suggested to be a primary cause of the decay of CXCL12 expression in several cancers, including colon cancer [10]. Illumina methylome arrays of 87 colon carcinomas (40 MSS and 47 MSI tumors) were used to investigate CXCL12 promoter methylation (Table 2). This cohort included 31 CIMP-High samples, 6 of which exhibited the MSS/CIMP-High phenotype. An unsupervised classification of the associated β-values delineated 2 groups with either a methylated or an unmethylated profile for 4 of the 9 CpG sites screened (cg 26718433, cg26267854, cg23407507, cg17267805) that all mapped near the transcription start site position (−185 to 0) of CXCL12 promoter. Across samples, (Figure 4, Table 3) 38/47 tumors (81%) of the MSI tumors and 14/38 (36%) of the MSS tumors were methylated at these sites, whereas these sites remained unmethylated in the remaining 19% of MSI and 64% of MSS tumors. Among the 87 samples, 2 CIMP+/MSS−/MSI- samples were methylated at the 4 CpG sites.

**Table 2:** Characteristics of the 87 carcinomas analyzed for methylome

Carcinomas		*n* = 87
***Mean age ± S.D***.		68,28 ± 12,2
***Men:Women***		54:33
***Localization***	Proximal colon	49
	Distal colon	38
***UICC classification***	In situ Tis	3
	I	10
	II	32
	III	26
	IV	16
***MSS/MSI/CIMP***		38/47/31

*SD*: standard deviation.

**Table 3:** CXCL12 methylation proportion according to MSI, MSS and CIMP status for each CpG sites in CXCL12 nearest and within the start of CXCL12 gene

CpG site Target ID	Position	dTSS	CpG Island according to UCSC	% methylated in Normal Tissues	% methylated in MSI tumors	% methylated in MSS tumors	*p*-value (diff % MSI vs %MSS)	% methylated in CIMP+ tumors	% methylated in CIMP- tumors	*p*-value (diff % CIMP+ vs %CIMP-	% methylated in MSI CIMP- tumors	% methylated in MSI CIMP+ tumors	% methylated in MSS CIMP- tumors	% methylated in MSS CIMP+ tumors	*p*-value (diff % MSI x CIMP)
cg11267527	44881934	−1389	Island	nd	100%	100%	-	100%	100%	–	100%	100%	100%	100%	–
cg00499822	44881551	−1006	Island	100%	91%	83%	0.37	94%	82%	0.26	92%	86%	80%	100%	0.55
cg18618334	44881054	−509	Island	4%	72%	33%	5.3E-04	90%	24%	2.1E-08	92%	21%	26%	80%	5.8E-08
cg26718433	44880730	−185	Island	nd	77%	30%	6.4E-05	94%	27%	7.0E-09	92%	43%	20%	100%	1.2E-08
cg26267854	44880562	−17	Island	nd	81%	43%	6.0E-04	97%	39%	1.2E-07	96%	50%	34%	100%	7.1E-07
cg23407507	44880559	−14	Island	nd	77%	33%	1.1E-04	94%	29%	2.1E-08	92%	43%	23%	100%	4.6E-08
cg17267805	44880545	0	Island	nd	81%	33%	4.3E-05	97%	31%	5.8E-09	96%	50%	23%	100%	8.6E-09
cg07001963	44879465	1080	N_Shore	nd	74%	33%	2.4E-04	97%	22%	2.0E-10	96%	21%	23%	100%	1.0E-09
cg12793525	44875989	4556	N_Shelf	nd	100%	100%	-	100%	100%	-	100%	100%	100%	100%	-

CpGisland (UCSC, chr10:44879714-44882391).

Because 46 of the 87 tumors analyzed for the methylation status of the CXCL12 gene were included in the expression data set [16], we thus examined the association between methylation and expression levels. Strikingly, these two variables did not correlate (correlation −0.22, *p* = 0.074; Supplementary Figure 1). In addition, there is no correlation between the level of CXCL12 expression and the methylation status of each individual CpG island analyzed.

Together, these data establish that the decreased expression of CXCL12 is an early and frequent event in human colon tumors which does not result from allelic loss. As considering that the CXCL12 promoter region is methylated in only a minority of MSS tumors, the promoter hypermethylation does not fully explain the silencing of the CXCL12 gene.

### Expression of CXCL12 in human colon cancer cells

#### Methylation status

As verified by MS-PCR, SW480, HCT116 and TC7 human colon cancer cells are characterized by different CpG island methylation profiles at the CXCL12 locus and at five additional genes, known to specify the methylator phenotype (Supplementary Table 1). Two of the three cell lines had a methylated promoter (HCT116 and TC7 cells). However, despite an unmethylated promoter, the SW480 cells did not express CXCL12.

#### Effect of histone deacetylase inhibitors

These observations suggest that the methylation of the promoter cannot by itself justify the loss of expression of CXCL12; thus we evaluated whether acetylation could participate to the loss of expression in the three colon cell lines.

**Figure 5:**
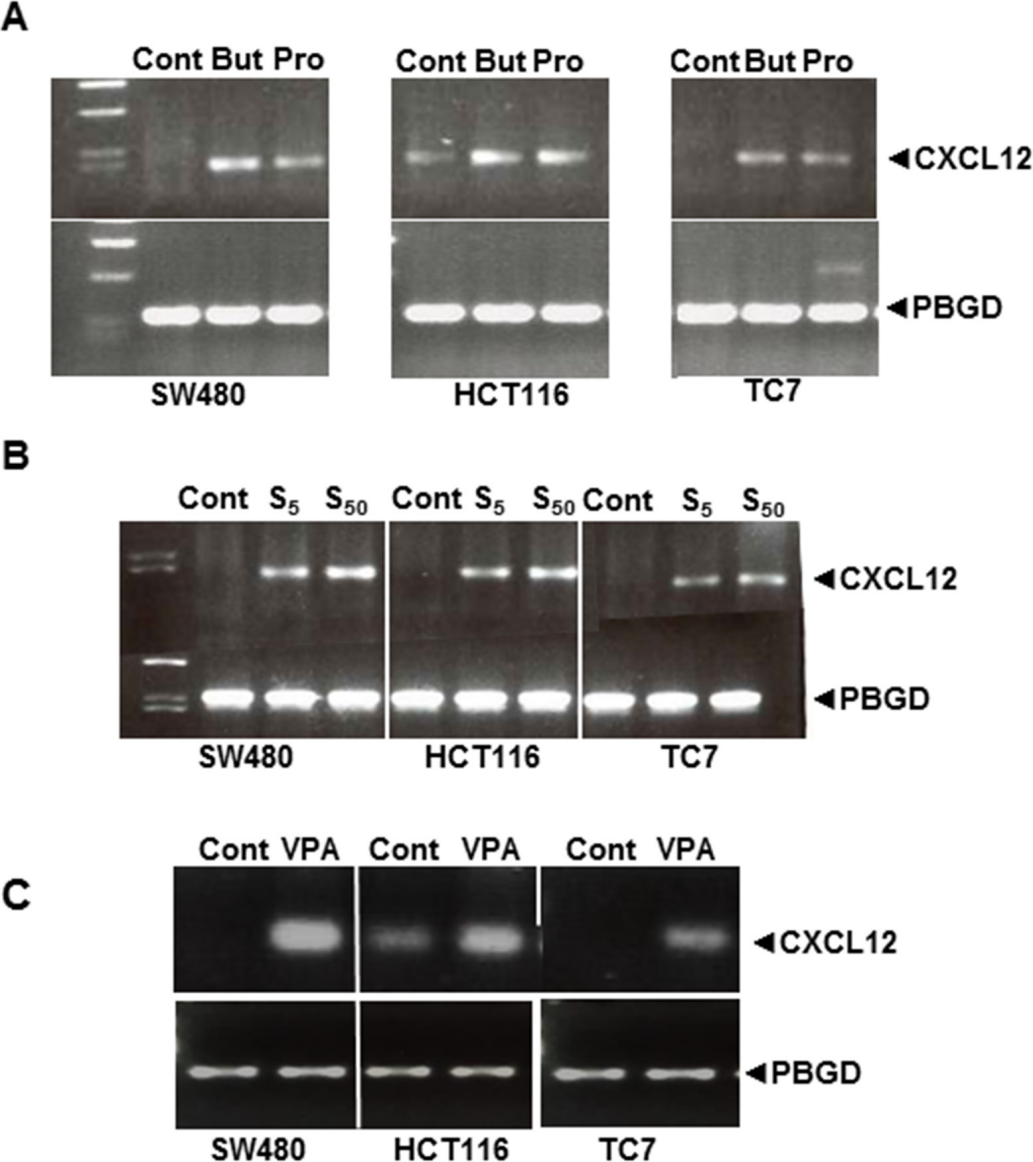
Strategies to re-express CXCL12 mRNA in SW480, HCT116 and TC7 cell lines after treatment for 24 h with (**A**) the natural short-chain fatty acid (SCFA) HDAC inhibitors Butyrate (10 mM) and propionate (15 mM) and (**B**) pharmacological HDAC inhibitors SAHA (S, 5 and 50 μM) and (**C**) VPA (1 mM). Results represent experiments run in triplicate with 4 wells per experiment.

Sodium butyrate (a short-chain fatty acid, SCFA) plays a major role in the modulation of gene expression via histone acetylation by inhibiting histone deacetylases (HDACs) [18]. Therefore, we tested the effects of butyrate and propionate, another SCFA, on CXCL12 expression in the three cell lines. Interestingly, low concentrations of both molecules could induce CXCL12 expression after 24 h of exposure (Figure 5A). We then tested the effects of two pharmacological HDAC inhibitors: the pan-HDAC inhibitor suberoylanilide hydroxamic acid (SAHA; 5 and 50 μM) and the class I/II HDAC inhibitor valproic acid (VPA; 1 mM). Both inhibitors stimulated CXCL12 mRNA expression in the three cell lines (Figure 5B–5C).

**Figure 6:**
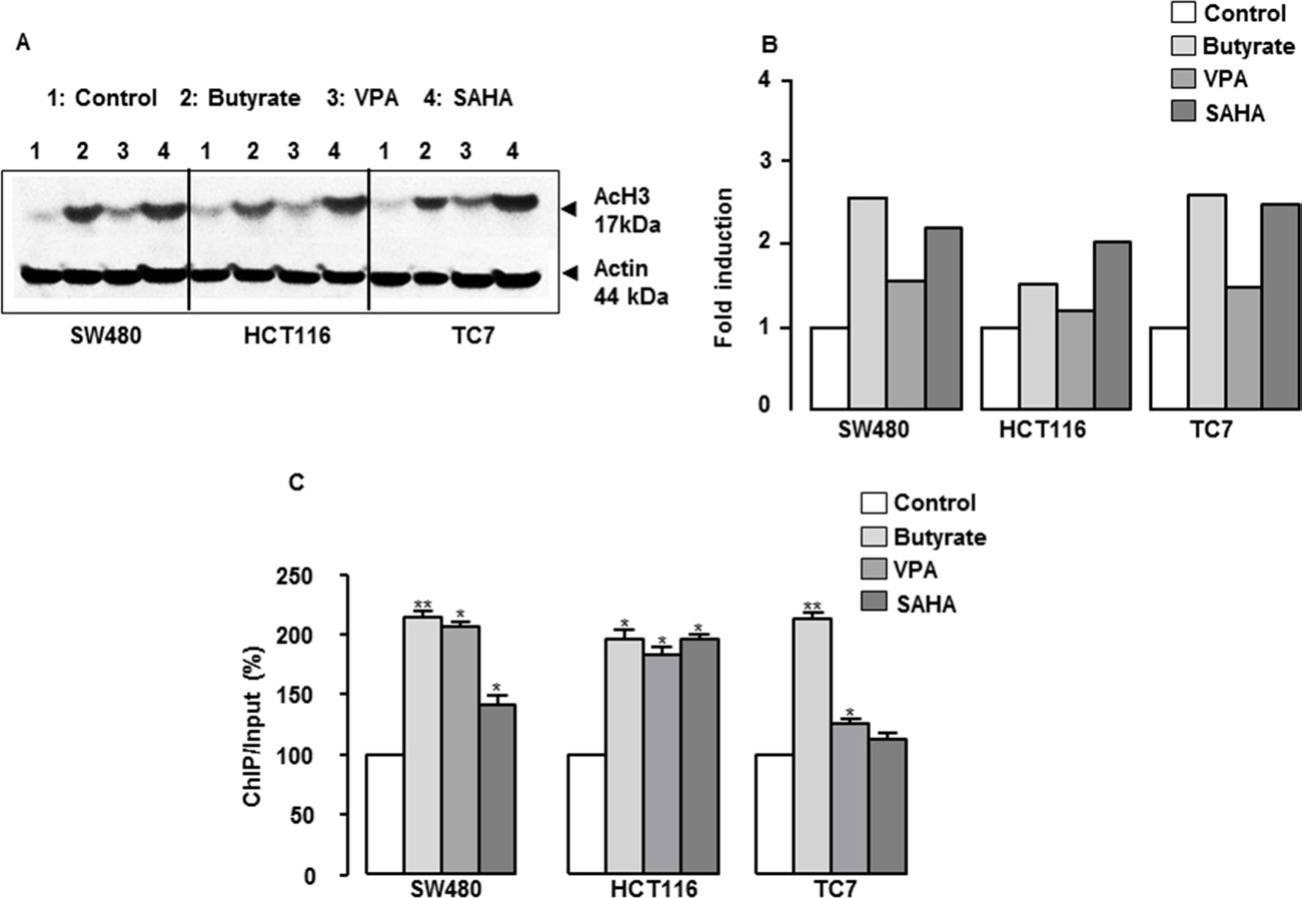
Role of histone acetylation in the regulation of CXCL12 gene expression and functional study (**A**) Representative experiment of butyrate- and VPA-induced histone H3 acetylation as quantified by western blotting in SW480, HCT116 and TC7 cell lines. (**B**) ChIP assays were performed on colon cells treated with but (10 mM) or VPA (1 mM) for 24 h, and the levels of the CXCL12 promoter in the immunoprecipitates were measured by RT-qPCR in SW480, HCT116 and TC7 cell lines.

The global acetylation of histone H3 (AcH3) was determined to investigate the status of histone acetylation after treatment with HDAC inhibitors, and the specific association of AcH3 with the CXCL12 promoter was examined using ChIP assays. As shown in Figure 6A–6B, butyrate, VPA and SAHA treatment increased the overall histone H3 acetylation in the three cell lines, butyrate and SAHA being the most potent compounds. In addition, the inhibitors increased the level of histone H3 N-terminal acetylation within the CXCL12 promoter (Figure 6C). Thus, HDAC inhibitors of different pharmacological classes restore and/or induce CXCL12 expression in human colon cancer cell lines.

#### Respective involvement of DNA methylation and histone acetylation in CXCL12 expression

To better define the respective roles of DNA methylation and histone acetylation on the expression of the CXCL12 gene, we quantified by RT-qPCR the ability of the different treatments (5-aza, SAHA, VPA and butyrate) to stimulate CXCL12 mRNA expression in the SW480 cell line the CXCL12 transcript was virtually undetectable in untreated cells (Ct > 45 cycles); our data show that prolonged and daily treatment with 5-aza had only minor effects (Relative Ratio RR = 0.0002), compared to SAHA (RR = 0.015), VPA (RR = 0.063) and butyrate, which reached a level similar to the one of normal human colon mucosa (RR = 0.13; Supplementary Figure 2). Similar results were observed for the two other colon cell lines (TC7 and HT29, data not shown). Combining 5-aza with any HDAC inhibitor did not further increase CXCL12 expression compared to the respective HDAC inhibitors alone (data not shown). Altogether, in our model of colon cancer cells, defect of histone acetylation would be a major epigenetic mechanism for CXCL12 downregulation, alone or in combination with promoter hypermethylation.

#### Involvement of PCAF loss in CXCL12 silencing

**Figure 7:**
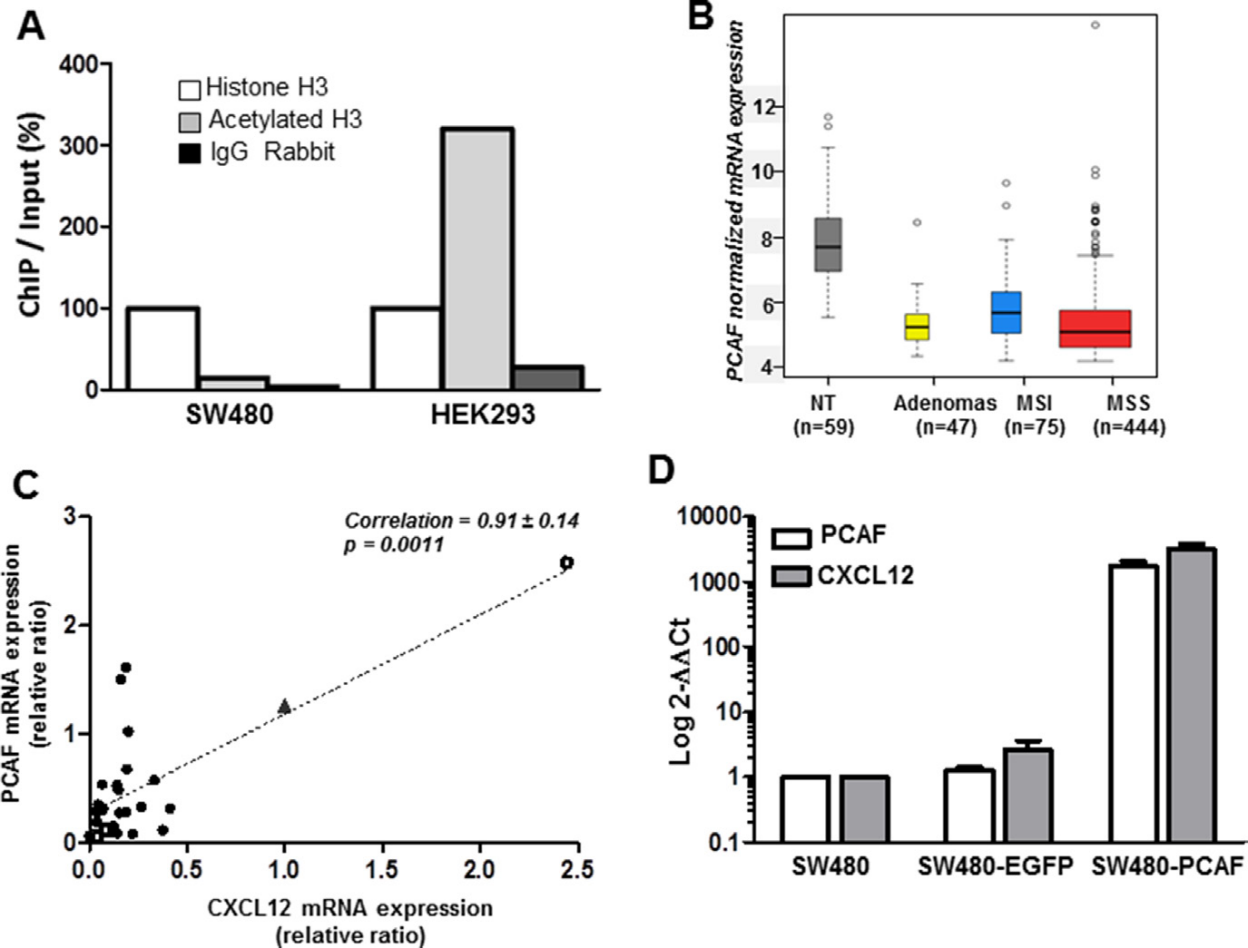
Histone H3 acetylation and PCAF expression (**A**) ChIP assays were performed on SW480 and HEK293 cell lines and the levels of the CXCL12 promoter in the immunoprecipitates were measured by RT-QPCR. (**B**) PCAF mRNA expression distribution. Boxplot of intensity values according to sample types (59 non-tumor tissues, 47 adenomas, 75 MSI carcinomas, 444 MSS carcinomas). (**C**) Correlation at the mRNA level (RT-qPCR) between CXL12 and PCAF expression in 26 colon tumor samples (black circles), a carcinoma expressing CXCL12 as in normal mucosa (open circle), two colon cancer cell lines (SW480 and HT29, open squares) and the human kidney embryonic cell line expressing CXCL12 (HEK293, black triangle). (**D**) Effect of PCAF forced-expression on CXCL12 in SW480 cells. PCAF and CXCL12 mRNA expression were evaluated 24 h after transfection with 3 μg of a control plasmid (SW480-EGFP) and a PCAF-expressing plasmid (SW480-PCAF). The mean Ct for CXCL12 and PCAF was normalized with PBGD and SW480 as controls and the results were expressed as 2^−ΔΔCt^. Experiments have been repeated in triplicate and results are representative of three sets of independent samples per group.

To further evaluate the histone H3 acetylation status in colon cancer, we performed chromatin immunoprecipitation (ChIP) experiments to compare the SW480 colon cancer cell line to HEK293, a human kidney embryo cell line that express CXCL12 at a high level. As shown in Figure 7A, we observed a significant higher proportion of acetylated H3 (ratio 20:1) in HEK293 as compared to SW480 cells.

Furthermore, since the treatment with HDAC inhibitors resulted only in partial CXCL12 re-expression and required a long-term exposure to the drugs, we hypothesized that an initial defect in histone acetylation could be, at least in part, responsible for the loss of CXCL12 expression, rather an increased deacetylation process. To confirm this hypothesis, we re-analyzed the most differentially expressed genes in our large transcriptomic data and focused on genes implicated in histone acetylation or deacetylation. Accordingly, we found a significant loss of the histone acetyltransferase (HAT) PCAF expression, in 72% of human colon cancer samples (Figure 7B, *p* = 3.17e^−54^). In contrast, there was no significant modification of the expression of histone deacetylase genes. As observed for CXCL12, loss of PCAF was found in adenomas as well as in MSS and MSI tumors. These results were further confirmed by qRT-PCR in a validation cohort of 26 patients and 3 colon cancer cell lines (Figure 7C. Supplementary Table 2). The expression of CXCL12 and PCAF was significantly correlated both in patients tumors, in a human kidney embryonic cell line expressing CXCL12 (HEK293) and two colon cancer cell lines not expressing CXCL12 (SW480 and HT29) (*p* < 0.001).

Finally we evaluated the effect of PCAF-forced expression in SW480 cell line after a transient transfection with a plasmid encoding the human PCAF gene. PCAF expression plasmid restored CXCL12 mRNA *in vitro* (Figure 7D). We observed a highly significant increase (over 1000-fold) of CXCL12 gene expression in the transfected cells (Figure 7D). This result provides an argument in favor of PCAF implication in CXCL12 expression regulation.

### Functional relevance of CXCL12 reexpression *in vitro*

#### Effect of CXCL12 reexpression on cell migration *in vitro*

**Figure 8:**
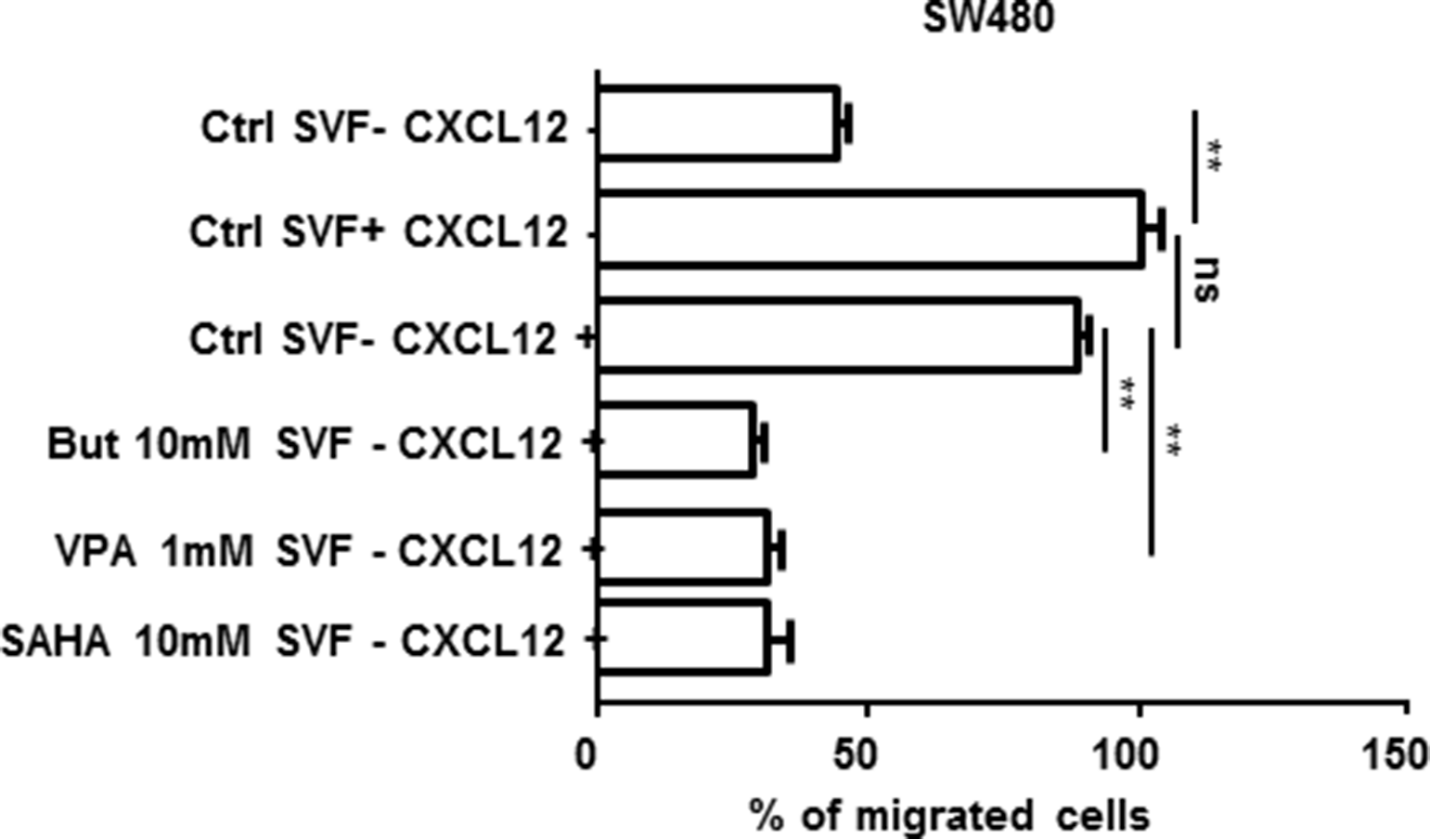
SW480 cell migration in Boyden chamber assays Cells remaining in the upper chamber were mechanically removed after 24 h of incubation w/o or with 10% fetal calf serum, 50 nM CXCL12 and/or 10 mM but or 1 mM VPA. The cells that had migrated to the lower chamber were counted after staining with the fluorescent dye DAPI (or 4′,6-diamidino-2-phenylindole). Quantification was performed by counting five random fields for each chamber under a microscope (Mann-Whitney parametric test, **p < 0.001; ns: non-significant).

The motility of SW480 cells was compared before and after treatment with 1 mM VPA, 10 mM butyrate or 10 mM SAHA in Boyden chambers. As illustrated in Figure 8, the addition of 50 nM CXCL12 to the bottom compartment stimulated the migration of the cells from the upper compartment. However, when the cells in the upper compartment were treated with 10 mM butyrate, 1mM VPA or 10 mM SAHA in order to reexpress CXCL12 (as measured by Elisa test, data not shown), the cells were no more attracted to the bottom compartment and therefore the migration was strongly compromised (about 65% inhibition; *p* < 0.001).

#### Effect of VPA treatment on tumor growth and CXCL12 re-expression in a xenografted human colon tumor

**Figure 9:**
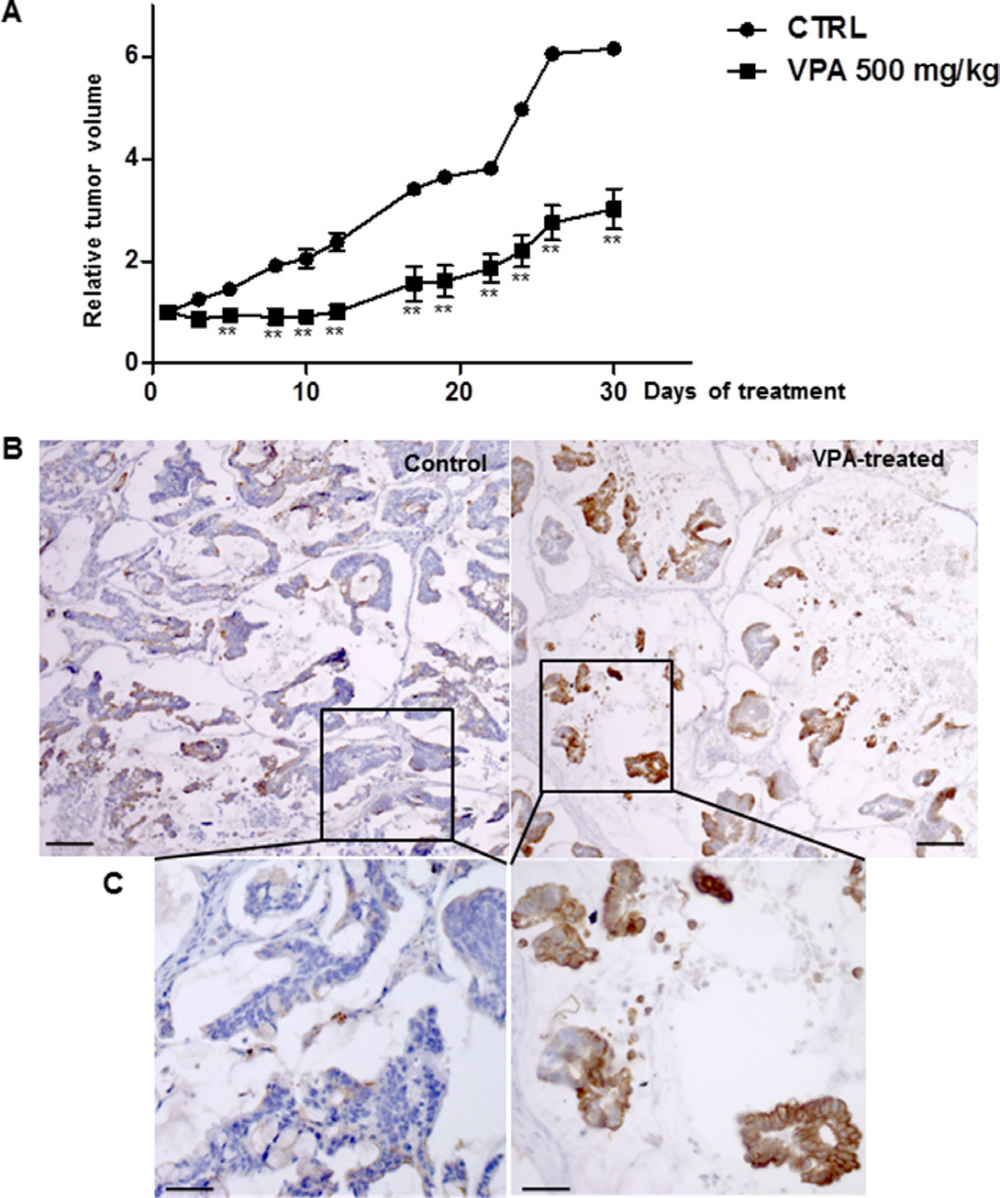
VPA treatment in a subcutaneous human colon tumor xenograft (**A**) Tumor growth was measured daily before and throughout the intraperitoneal administration of VPA (500 mg/kg) for 30 days. Data represent the relative tumor volume plotted as a function of time. Five mice, each bearing two individual tumors, were included in each group. Mann-Whitney parametric test, **p < 0.02. (**B**) Immunohistochemical CXCL12 expression in human colon cancer xenografts treated with 500 mg/kg VPA for 30 days and control (untreated) xenografts. (**C**) Enlargement of a part of the pictures shown in B. The results represent five mice per group.

The impact of HDAC inhibition on tumor growth was evaluated *in vivo* in xenografted human colon tumors treated (*n* = 5) with 500 mg/kg VPA for 30 days. The control group (*n* = 5) only received vehicle. The VPA treatment resulted in a 50% decrease in tumor volume (Figure 9A) with extensive necrosis and few residual tumor cells (data not shown). As expected, CXCL12 expression was almost absent in the tumor cells of the untreated mice while we observed that CXCL12 expression was restored in the residual tumor cells after VPA treatment (Figure 9B–9C).

#### Effect of VPA treatment on tumor formation and CXCL12 expression in APC mutated mice

**Figure 10:**
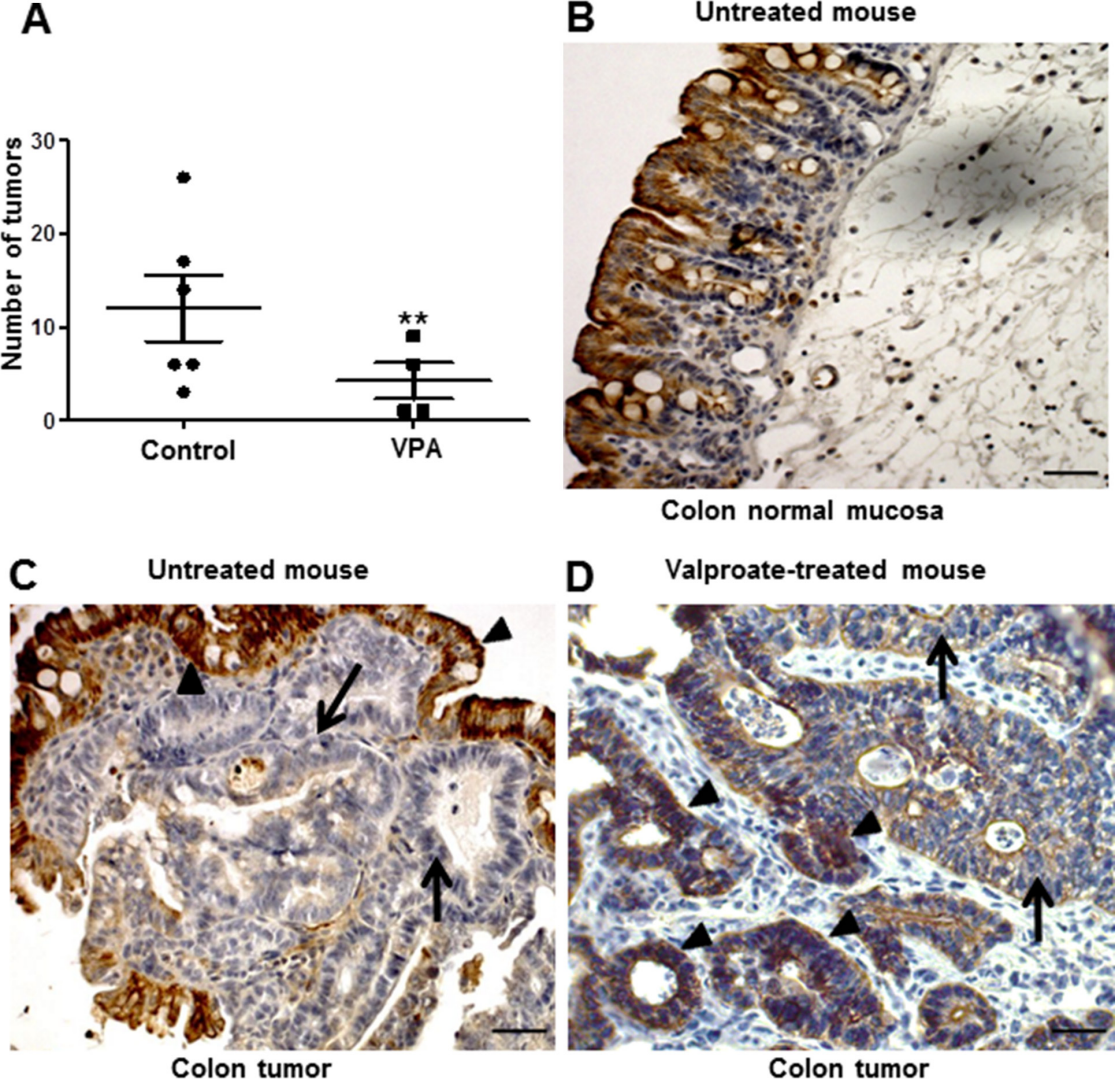
Intestinal CXCL12 expression and carcinoma development in control and VPA-treated APC^Δ14/+^ mice (**A**) Quantitative evaluation of the number of carcinomas in the entire intestinal tract of control and VPA-treated APC^Δ14/+^ mice. The APC^Δ14/+^ mice were intraperitoneally injected with PBS (*n* = 6) or VPA (*n* = 5; 500 mg/kg/day) for 30 days. Mann-Whitney parametric test, **p < 0.001. (**B**–**D**) Immuno-histochemical CXCL12 expression in the normal colon mucosa of control APC^Δ14/+^ mice (PBS treated, (B) in a VPA-untreated tumor of an APC^Δ14/+^ mouse (C) and in the colon of APC^Δ14/+^ mice treated with 500 mg/kg VPA for 30 days (D).

Next, we evaluated the effect of HDAC inhibition and CXCL12 re-expression on tumor initiation/maintenance in the APC^Δ14/+^ mouse model. To this end, APC^Δ14/+^ mice were administered VPA (500 mg/kg) or PBS for 30 days. As shown in Figure 10A, a daily injection of VPA for 30 days significantly reduced the number of intestinal tumors by 65%, both in small intestine and colon (*p* < 0.001). A univariate negative binomial model indicated a sex effect (*p* = 0.012) and treatment effect (*p* = 0.014). Immunohistochemistry did not indicate CXCL12 expression in the tumor cell areas in the control group, whereas CXCL12 expression was patchy when re-expressed in the tumor cells of the VPA-treated group (Figure 10B–10D, arrows).

## DISCUSSION

Our study presents data obtained from a large retrospective cohort of samples of human carcinomas and adenomas. The results clearly confirm previous work [10] showing that a loss of CXCL12 expression is a hallmark of colon cancer and in addition, we demonstrate for the first time, that the loss occurs early in the colon pathogenesis process as it is already present in the adenomas. Moreover, two mouse models of chemically-induced and spontaneous intestinal cancer recapitulate the observations made in humans, which reinforces the aforementioned observations. However, although we confirm that in this large cohort, the CXCL12 gene promoter is a target for methylation [10] particularly in CIMP+ samples, our functional *in vitro* and *in vivo* studies clearly demonstrate that a defect of CXCL12 promoter histone acetylation may represent an additional process participating in CXCL12 expression extinction. Interestingly, the two carcinomas subtypes C4 and C6 which have higher CXCL12 expression levels than the four other subtypes – but still lower than in the normal mucosa - and that are associated with a worse prognosis for patients, aggregate downregulated genes of proliferation pathways and upregulated genes of migration/epithelial-mesenchymal transition pathways [16]. We might thus hypothesize that the reduction of CXCL12 expression might participate to the colon mucosa transformation early in the colon carcinogenesis process, and thereafter, according to the remaining level of CXCL12, the dissemination process would be facilitated.

### How does CXCL12 gene silencing impact tumor initiation and/or progression?

Colonic epithelial homeostasis based on bottom-up cell migration from the crypt base depends on multiple regulatory pathways, including E-cadherin/Beta-catenin interactions. Furthermore, the migration of enterocytes is reduced in several transgenic mouse models that display altered adherens junction protein expression, which may lead to adenoma formation [19]. The CXCL12 gradient along the crypt axis participates in the regulation of the continuous renewal of the colon epithelium and maintains the epithelial barrier integrity. Promoter acetylation, which could be sustained by the action of HDAC inhibitors such as butyrate, may be one of the processes that maintain a proper level of CXCL12 expression. Interestingly, a butyrate proximal-to-distal luminal gradient has been described in the normal colon epithelium and was also observed for CXCL12 expression in the present study [20, 21]. We showed that epigenetic modifications, including defects in promoter histone acetylation in MSS carcinomas and DNA methylation in MSI samples, decreased CXCL12 expression in tumor tissue.

For the first time, we identified PCAF, a lysine acetyl transferase enzyme that is related to apoptotic cell processes [22], as a master regulator of CXCL12 expression *in vitro*. We also demonstrated that loss of PCAF expression is an early event in colon cancer initiation, observed at the adenoma stage and maintained in carcinomas that correlates with CXCL12 silencing in human tumors. Loss of PCAF has already been described in pancreatic cancer, endometrial tumors or head and neck carcinomas, and has been implicated in different molecular processes regulating tumor initiation and/or progression [23–25]. Here we also show that forced-expression of PCAF in SW480 non-CXCL12 expressing cells, is a powerful inducer of CXCXL12, providing further evidence that in addition to methylation, level of promoter acetylation may participate to regulate CXCL12 expression in colon cancer cells.

A recent paper indicated that in cervical cancer cells, epigenetic reactivation of CXCL12 was observed when cells are treated with both methylation-inhibiting and acetylating agents, suggesting that in cervical cancer also, methylation and histone acetylation may participate to control the CXCL12 expression level [26].

### CXCL12 as a molecular target in colon cancer

Our results suggest that epigenetic-based therapeutic intervention, especially HDAC inhibition might be a powerful strategy. Clinically, it is easier to consider treatments that inhibit enzymatic activity rather than activating it. In cancer, the modulation of histone acetylation has been extensively studied, and many pan-HDAC inhibitors have recently been developed [27]. In our study, treatment with the natural SCFA butyrate *in vitro* most strongly restored CXCL12 expression compared with two pharmacological HDAC inhibitors, SAHA and VPA. Nevertheless, VPA could reduce ectopic tumor growth *in vivo* as well as the number of intestinal tumors in APC mutant mice, and this effect was concomitant to the re-expression of CXCL12. Colon cancer cells forced to endogenously express CXCL12 undergo anoïkis [28], whereas non-producing cells exogenously exposed to CXCL12 survived and migrated [29]. Although CXCL12 is the main effector of the changes seen *in vitro* in cell migration, or in the animal models, HDAC inhibition will bring about broad-ranging alterations in gene expression of which the increased expression of CXCL12. Nonetheless, these findings suggest that CXCL12 maintenance or re-expression by colon epithelial cells could have a preventive and/or therapeutic interest.

Numerous epidemiological and experimental studies have identified a correlation between a high-fiber diet and a decreased incidence of colon cancer [30]. Thus, these protective effects could be provided by butyrate, which is produced from the fermentation of non-digestible substrates by the enteric microbiota. Butyrate has been shown to inhibit the growth of colon carcinoma cells in several *in vivo* [31–32] and *in vitro* models [33], and this butyrate-induced cell growth inhibition and differentiation was partially due to the capacity of this SCFA to induce histone hyperacetylation via HDAC inhibition [34]. A recent report confirms a molecular mechanism by which butyrate functions as an HDAC inhibitor in cancer colonocytes [35]. Because several HDACs are overexpressed in early colon field carcinomas [36] and patients with colorectal carcinomas exhibit a significant reduction of butyrate-producing bacteria in the gut microbiota [37], cancer risk may correlate with changes in the colonic milieu, such as a depletion of butyrate producers and an increase in opportunistic pathogens with oncogenic potential.

In conclusion, besides confirming a major decrease of CXCL12 expression in carcinomas as already shown in colon cancer, we demonstrate that the decrease is a precocious event occurring already in adenomas. Regarding the CXCL12 loss, it results from a promoter acetylation defect for the large majority of carcinomas and treating APC mutant mice with a HDAC inhibitor importantly reduces the intestinal tumor development. Although the mechanistic link between CXCL12 silencing, the default promoter histone acetylation and colon cancer formation and progression require further investigation, the results of our study are of potential clinical interest.

Agents that promote or maintain histone acetylation through HDAC inhibition and/or HAT stimulation may help to lower colon adenoma/carcinoma incidence, especially in high-risk families, or could be included in therapeutic protocols to treat advanced colon cancer.

## MATERIALS AND METHODS

### Patients and tumor samples

The CXCL12 and PCAF gene status were evaluated based on an Array-Based Comparative Genomic Hybridization Analysis established from 7 national cohorts [16] that comprise 566 samples, which included 444 microsatellite-stable (MSS), 75 microsatellite-instable (MSI) and 47 adenomas. For the relative quantitative CXCL12 expression (RT-qPCR) and immune-histochemical labelling, 46 MSS carcinomas, which also belonged to the 444 MSS cohort, and 30 MSS adenomas were collected at the Biological Resources Center at the Strasbourg University Hospital (local cohort, Table 1) using protocols approved by the CRB institutional review. All patients provided written informed consent and were recruited into the study after it had undergone institutional review by the French Ethics Committee of Strasbourg. MSI or MSS phenotypes were characterized using a panel of five different microsatellite loci from the Bethesda reference panel. Tumors were characterized as MSI if two or more of the five markers showed microsatellite instability and MSS if none of the five markers showed instability. CIN was defined from CGH profiles and samples with at least 20% gain or loss of whole chromosomes or fractions of chromosomes [16].

The correlation between CXCL12 and PCAF expression at the mRNA level (RT-qPCR) was analyzed in an independent validation cohort of 26 MSS carcinomas (Supplementary Table 2).

### Transcriptome analysis

CXCL12 mRNA expression was evaluated in a gene expression data set of 519 MSS and MSI colon carcinomas [16], 47 adenomas (32 from GSE8671, 15 from GSE4183) and 59 non-tumor tissue samples (19 from GSE33582, 32 from GSE8671 and 8 from GSE4183). Thirty additional carcinomas from the GSE4183 data set were also integrated. The three data sets were normalized together by RMA normalization (R package affy). An unsupervised Gaussian mixture model clustering approach [38] (R package mclust) was used to define the best number of sample groups for CXCL12 expression data. Associations with annotations were assessed by an ANOVA, *t*-test or Fisher's exact test depending on the type of variable tested (R package stats).

### Methylome analysis

Whole-genome DNA methylation was analyzed in a subset of 87 tumors (Table 2) using the Illumina Infinium HumanMethylation450 Beadchips. Hybridizations were performed at Integragen (www.integragen.com) according to the manufacturer's specifications (Illumina). The chips were scanned using the Illumina HiScan SQ scanner, and the raw image data were imported into the GenomeStudio (v2011.1) methylation module (v1.9.2.). Only CXCL12 CpG sites within CpG islands were considered, as reported in Illumina annotations; sites with probes that contained a SNP were removed. Unsupervised hierarchical clustering, Ward linkage and Euclidian distance were performed to identify samples with different methylation profiles for these sites. For each site, β-values were assigned to the methylated/unmethylated status using an unsupervised mixture modeled fits approach (R package mclust). When no group was found, the site was considered methylated for all samples if the median of the β-values was below 0.3; otherwise, the site was considered unmethylated. The association of the methylation status with annotations was assessed with Fisher's exact test.

### Cell culture and treatments

Human colon carcinoma HCT116, SW480 (obtained in 2013 from the American Type Culture Collection, Manassas, VA, USA; experiments started at P15 and P70 for HCT116 and SW480 respectively) and TC7 (kindly provided by M. Rousset), [39] cells were maintained at 37°C in standard culture conditions. The mutational status of BRAF, PI3K and KRAS has been validated as previously shown [40]. Cells were treated during exponential growth 30% confluence with 5-azacytidine at 2.5 μM Vidaza^®^, 25 mg/ml (Celgene) every day for up to 10 days; with sodium butyrate at 10 mM 110.09 g/mol (Sigma), sodium propionate at 15 mM 96.06 g/mol (Sigma), SAHA Vorinostat at 5 and 50 μM 264.32 g/mol (Sigma) or Valproic Acid at 1 mM during 24 hours 166.19 g/mol (Sigma). Experiments were run in triplicate with *n* = 4 wells in each experiment.

### PCAF-Expressing SW480 cells

Cells (500.10^6^) were transfected with 3 μg of the pMSCVpuro-PCAF plasmid (Addgene, #63705) encoding the human PCAF gene with JetPEI (Polyplus Transfection), according to manufacturer's instructions. The DNA mix was incubated with the JetPEI for 20 min at room temperature. After incubation, the complex was added to the culture medium. As control, we transfected the cells with the control vector pEGFP-C1 (Addgene, #U55763). The transfection efficiency was confirmed after 24 h by quantitative RT-PCR.

### Cell migration assay

Single cell migration assay was performed in Boyden Chambers BD (Biosciences) with the upper and lower compartments filled without and with 50 nM CXCL12 cell culture medium respectively, with or without HDAC inhibitors (1 mM valproic Acid or 10 mM butyrate, 24 h treatment before migration assay). After 12 h of migration in the chamber, the cells were fixed with 4% PFA, stained with DAPI and counted under the microscope.

### Methylation specific PCR

Methylation patterns in the CpG islands of CXCL12 and of 5 methylation markers (RUNX3, SOCS1, NEUROG1, CACNA1G, IGF2) were determined by methylation specific PCR, and the CIMP+ phenotype was defined by CpG island promoter methylation of at least 3 out of these 5 markers [41]. Multiplex PCR allowed amplification of methylated and unmethylated fragments in the same PCR reaction and were analysed by capillary electrophoresis on a 3130 Genetic Analyzer (Applied Biosystems, Forster City, CA, USA). *In vitro* methylated DNA (CpGGenome^™^ Universal Methylated DNA, MP Biomedicals, Irvine, USA) was used as a positive control for the methylated alleles, and unmethylated DNA was used as a negative control. Moreover, a control of the sensitivity of detection of methylated DNA (5% methylated DNA + 95% unmethylated DNA) was also included in each experiment.

PCR amplifications were performed in a final volume of 50 μl consisting of 0.2 μM of each primer (except for IGF2 0.1 μM and CXCL12 0.75 μM), 2.5 mM MgCl_2_, 1M Betaine, 70 μM of each dNTP, Taq Gold Buffer 1 ×, 5 units Taq Gold and ~ 10ng of bisulfite converted DNA. After an initial denaturation step at 95°C for 10 min, 33 cycles were performed at 94°C for 45 sec, 60°c for 90 sec and 65°C for 25 sec, followed by a final elongation step at 65°C for 30 min. The primers for the MS-PCR are listed in (Supplementary Table 3).

### Relative quantitative RT-PCR

mRNA expression of the human CXCL12, PCAF and control HMBS genes was evaluated by relative quantitative real-time PCR (RT-qPCR) analysis using the LightCycler system (Roche Molecular Biochemicals) and FastStart DNA Master Mix SYBR Green I (Roche Diagnostics) as previously described [42].

### Chromatin immunoprecipitation assay (ChIP assay)

The ChIP protocol was adapted from MilliporeChIP Assay Kit (17-295) with minor modifications, as previously described [43]. In brief, trypsinized cells were washed 2× in PBS and 1.2 × 10^7^ cells were cross-linked at 37°C for 10 min in 5 ml PBS/0.5%BSA/1% ultra-pure formaldehyde (Electron Microscopy Sciences). Quenching with 125 mM glycine and a cold PBS wash (containing 1x protease inhibitor cocktail (PIC); Roche) was followed by cell lysis in 5 ml of 1% Triton X-100, 50 mM MgCl2, 100 mM Tris-HCl (pH 7.1), 11% sucrose, 1 × PIC for 10 min on ice. Nuclei were pelleted at 2,000 rpm for10 min at 4°C and were lysed in 500 μl of 1% SDS, 50 mM Tris-HCl, 10 mM EDTA, 1 × PIC. Chromatin was sonicated to 800–300 bp using Bioruptor 200 (Diagenode), cleared by centrifugation and sonication efficiency was verified. Sonicated chromatin diluted 4× with 0.01% SDS, 1.1% Triton X-100, 1.2 mM EDTA, 16.7 mM TRIS-HCl pH 8.1, 167 mM NaCl, 1 × PIC was pre-cleared by rotating for 1 h at 4°C with50 μl Magna ChIP Protein A Magnetic Beads (Millipore 16-661) previously blocked with 0.5% BSA for 1h at 4°C. 3 × 10^6^cell equivalents were further diluted 2.5× in the same buffer, and incubated overnight with 5 μg anti-AcH3 (N-Term specific, Millipore 06-599), 5 μg anti-H3 (Millipore 06-755) and 5 μg rabbit IgG (Millipore 12-370). Protein-DNA complexes were bound to 30 μl Protein A Magnetic Beads for 5–6 h at 4°C and washed 1× each with low-salt buffer (20 mM Tris-HCl, pH8.1, 150 mMNaCl, 2 mM EDTA, 1%Triton X100, 0.1% SDS), high-salt buffer (20 mMTris-HCl, pH8.1, 500 mMNaCl, 2 mM EDTA, 1%Triton X100, 0.1% SDS), LiCl buffer (10 mMTris-HCl, pH8.1, 1 mM EDTA, 1% deoxycholate, 1% NP40, 0.25 M LiCl), and TE. Samples were further processed using iPure Kit (Diagenode) according to the manufacturer's instructions. DNA recovered after ChIP was used for PCR detection of CXCL12 promoter and a nonrelated region in the CXCL12 exon 2 as a control (see the primer sequences in Supplementary Table 4). ChIP-qPCR data were analysed relative to total histone H3 1% input adjusted to 100%, fold enrichment was calculated according to the 2^−ΔΔCt^ method and PCR efficiency was calculated after serial dilutions of standard sample (slope : -3,25; Efficiency : 103%).

### Western blot analysis

Whole cell extracts prepared from HCT116, SW480 and TC7 colon cancer cell lines were separated on a 12% SDS-PAGE gel and subjected to immunoblot analysis. Primary antibody for Western Blot analysis was AcH3 (5 μg/ml Millipore) and anti-actin (1/15000, Millipore^®^). Protein expression was detected by using horseradish peroxidase-conjugated goat anti-mouse or anti-rabbit secondary antibodies (Amersham) with ECL reagent (Amersham) following the manufacturer's instructions.

### Human tumor xenografts, mice models and treatments

Six- to eight-week-old female athymic nude mice (NMRI-Foxn1nu/Fox1nu, Janvier^®^) were used, and the procedures were approved by the French Ethical Committee (Cremeas, N°AL/85/92/02/13). Human tumor tissue fragments were obtained in accordance with the ethical standards of the institutional committee. Cancer tissues were injected in the flanks of the mice [17]. The mean RTV was calculated for each group.

Wild type, APC Min^+^, APC^Δ14/+^ mice (C57BL/6 (B6) background) were housed according to the guidelines of the Ethic Committee of the University of Strasbourg.

Wild-type mice were treated with 10 mg/kg azoxymethane as previously described [44]. For valproate treatment (VPA), 16- to 18-week-old heterozygous APC^Δ14/+^ mice [45] were intraperitoneally injected with PBS (*n* = 6) or VPA (*n* = 5; 500 mg/kg/day) for 30 days. At the end of the treatment, the intestinal tracts were fixed in 4% PFA in PBS for immunohistochemistry, and the frozen samples were kept at −80°C for molecular analyses.

### Immunohistochemistry

Immunohistochemistry used standard procedures with tumors fixed zinc fixative and embedded in paraffin. Tissue sections were incubated with the primary antibodies against CXCR4 (1:200; eBiosciences) and CXCR7 (1:75; ThermoScientific) after antigen retrieval and immunostaining was developed with a liquid DAB substrate kit (Roche).

### Statistical analysis

The data were analyzed with the Mann-Whitney parametric test, and the significance level was set to 5%. To evaluate the effect of VPA on APC Δ14/+ mice, the macroscopically detected tumors were counted in the control and treated animals. A negative binomial model was fitted, and a *p*-value < 0.05 was considered significant. The model was run under R 3.0.2. with the MASS package “glm.nb” function.

## SUPPLEMENTARY MATERIALS FIGURES AND TABLES


