# Supplementary material for: Histone hypoacetylation contributes to CXCL12 downregulation in colon cancer: impact on tumor growth and cell migration
Source: Oncotarget. 2017 Mar 17;8(24):38351–66. doi: 10.18632/oncotarget.16323 (PMC5503537; doi:10.18632/oncotarget.16323)
Supplement: Supplementary file 1 [file oncotarget-08-38351-s001.pdf]

Histone hypoacetylation contributes to CXCL12 downregulation in colon cancer: impact on tumor growth and cell migration

Supplementary Materials

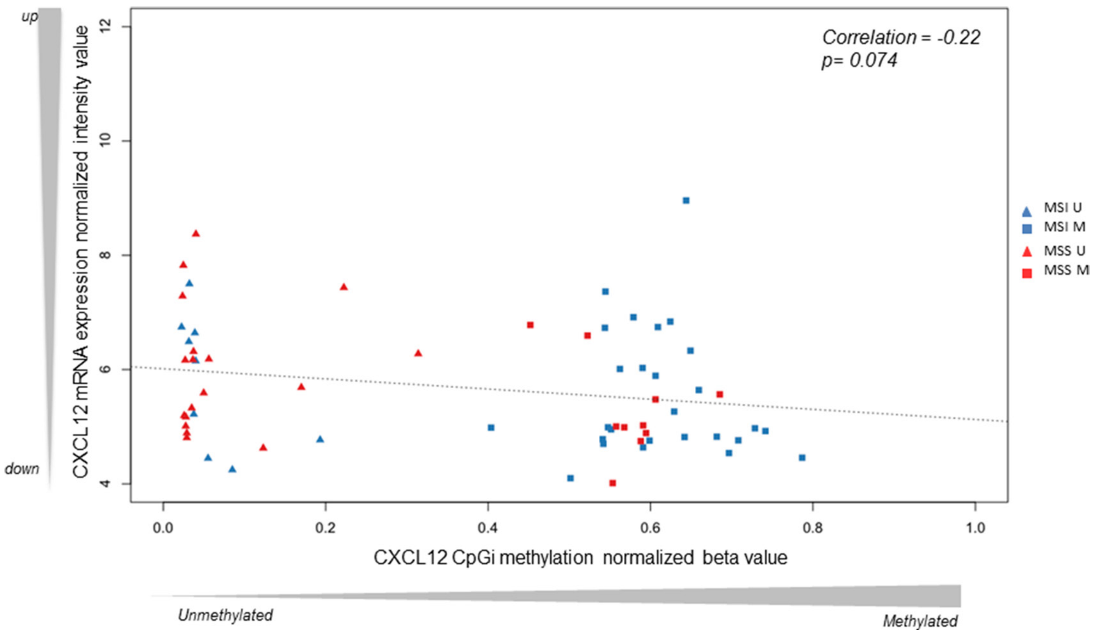

Supplementary Figure 1: Correlation between CXCL12 promoter methylation and mRNA expression. y-axis corresponds to Affymetrix log2 normalized intensity values of CXCL12 probe set 209687_at and x-axis to β-values of the CpG site cg17267805.

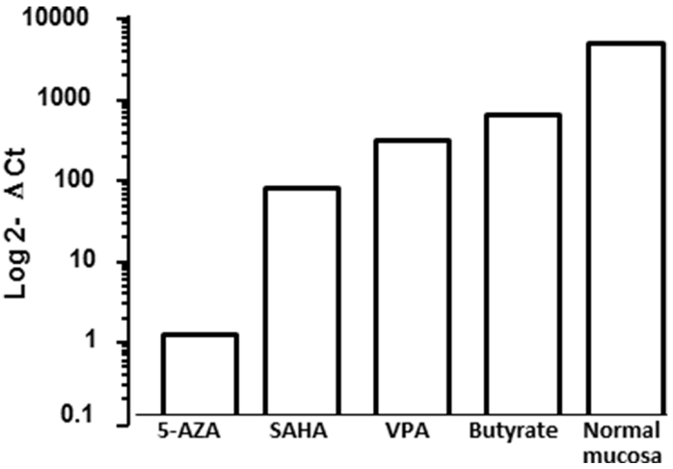

Supplementary Figure 2: Respective roles of methylation and acetylation in CXCL12 mRNA reexpression. SW480 cells were treated with 5-AZA (2.5 µM), SAHA (50 µM), VPA (1 mM) and butyrate (10 mM) for 24 h. The mean Ct for CXCL12 was calculated for each treatment, normalized with PBGD as control and the results were expressed as 2-ΔCt. Experiments have been repeated in triplicate. Results are representative of three sets of independent samples per group.

Supplementary Table 1: CIMP profile and CXCL12 methylation in colon cancer cells

	CACNA1G	IGF2	NEUROG1	RUNX3	SOCS1	CXCL12	CIMP
**HCT116**	M	M	M	U	M	M	**CIMP-High**
**SW480**	U	U	U	U	U	U	**CIMP-Low**
**Caco2/TC7**	U	U	M	U	M	M	**CIMP-Low**

M = methylated; U = Unmethylated.

Supplementary Table 2: Characteristics of the 26 carcinomas of the validation cohort for PCAF and CXCL12 expression

**Carcinomas**		***n* = 26**
Mean age ± S.D	67 ± 12	
Men:Women	15/11	26
Localization	Proximal colon	8
	Distal colon	18
UICC classification	In situ Tis)	
	I	2
	II	10
	III	8
	IV	6
MSS/MSI/CIMP	26/0/0	26

Supplementary Table 3: Primer sequences for MS-PCR

PRIMERS		SEQUENCES
CACN1G	CACN1G-UF	FAM- GTTTTGGTTTTTTTGTTTTGTGT
	CACN1G-UR	CACCCTCTCAAAACAACTTCAC
	CACN1G-MF	FAM - TCGGTTTTTTCGTTTCGCGT
	CACN1G-MR	CTCTCGAAACGACTTCGCCG
IGF2	IGF2-UF	VIC- TTGGAGTGGTTTTGGTGTTGT
	IGF2-UR	CAAACCACAAACACCCAACTCAA
	IGF2-MF	VIC-GCGGTTTCGGTGTCGTTATC
	IGF2-MR	CGCGAACGCCCAACTCGA
NEUROG1	NEUROG1-UF	FAM-TATTGTTGGTTAATTGGTGGTG
	NEUROG1-UR	ACATACCTCAACCACTAATCAC
	NEUROG1-MF	FAM-TCGGTTAATCGGCGGCGTC
	NEUROG1-MR	TACCTCGACCGCTAATCGC
RUNX3	RUNX3-UF	NED-GTTTATGGGAATATGTATAATAGTGG
	RUNX3-UR	CCCACTTCTCACAACAACAACA
	RUNX3-MF	NED-CGGGAATACGTATAATAGCG
	RUNX3-MR	GCTTCTCGCGACAACGACG
SOCS1	SOCS1-UF	PET-TATTTTTTTGGTGTGTGATAGTTG
	SOCS1-UR	AAACAACCAACCTAAAAATACACA
	SOCS1-MF	PET –ATTTTTTTGGTGCGCGATAGTC
	SOCS1-MR	GACCGACCTAAAAATACACGC
CXCL12	CXCL12-UF	FAM-AAGGTTGGAGTGTATTGTGTTTTG
	CXCL12-UR	AAAACCAAATACTAACCATAACCA
	CXCL12-MF	FAM-CGGAGCGTATTGCGTTTC
	CXCL12-MR	CCGAATACTAACCGTAACCG

Supplementary Table 4: Primer sequences for chromatin immunoprecipitation

	Primer sequences (5′-3′)
CXCL12-F	CGCCTAAGGTCCTCAGTCTC
CXCL12-R	CTCCTCCGCTCCCTCTGT
CXCL12 Exon 2-F	GGAAGCCCGTCAGCCTG
CXCL12 Exon 2-R	ACAATCTGAAGGGCACAGTTTG
